# Inadequate Intake of Energy and Nutrients Is Common in Older Family Caregivers

**DOI:** 10.3390/nu13082763

**Published:** 2021-08-12

**Authors:** Sohvi Koponen, Irma Nykänen, Roosa-Maria Savela, Tarja Välimäki, Anna Liisa Suominen, Ursula Schwab

**Affiliations:** 1 Institution of Public Health and Clinical Nutrition, School of Medicine, University of Eastern Finland, P.O. Box 1627, FI-70211 Kuopio, Finland; irma.nykanen@uef.fi (I.N.); ursula.schwab@uef.fi (U.S.); 2 Department of Medicine, Endocrinology and Clinical Nutrition, Kuopio University Hospital, P.O. Box 100, FI-70029 Kuopio, Finland; 3 Department of Nursing Science, University of Eastern Finland, P.O. Box 1627, FI-70211 Kuopio, Finland; roosa-maria.savela@uef.fi (R.-M.S.); tarja.valimaki@uef.fi (T.V.); 4 Institution of Dentistry, School of Medicine, University of Eastern Finland, P.O. Box 1627, FI-70211 Kuopio, Finland; liisa.suominen@uef.fi; 5 Department of Oral and Maxillofacial Diseases, Kuopio University Hospital, P.O. Box 100, FI-70029 Kuopio, Finland

**Keywords:** nutritional status, malnutrition, dietary intake, family caregiver, older people

## Abstract

The aim of this study was to investigate the nutritional status, determinants of nutritional status, and adequacy of energy and nutrient intake of older family caregivers (FC). Nutritional status was measured using the Mini Nutritional Assessment (MNA), plasma albumin, plasma pre-albumin, and blood hemoglobin concentrations. Dietary intake was assessed with a three-day food record. Comorbidity (B −0.283, 95% CI: −0.492, −0.073), quality of life (B 0.045, 95% CI: 0.018, 0.072) and energy intake (B 0.001, 95% CI: 0.000, 0.002) were significantly associated with the MNA scores of the older FCs (*n* = 125). It was common for FCs to have lower than recommended intakes of energy and several nutrients, independent of the risk of malnutrition assessed by the MNA. Over half of the FCs had inadequate intake of protein, vitamin A, folate, and fiber, and 25–40% of the FCs had a low intake of vitamin D, vitamin E, thiamine, magnesium, iron, and selenium. It is important to follow both the nutritional status and dietary intake of older FCs regularly to find those with lower than recommended nutrient intake and to avoid poor nutritional status and its adverse effects hampering their ability to serve as FCs.

## 1. Introduction

Older family caregivers (FC) have a remarkable responsibility to take care of their older care recipients’ (CRs’) nutritional needs as well as CRs’ other care needs at home. The nutritional status of older institutionalized and home-care people has been widely studied. However, home-dwelling people, and especially FCs, are underrepresented, and there are only few studies about the nutritional status of FCs. These studies have shown that a large number of FCs are malnourished or at risk of malnutrition [1,2,3], and prevalence of malnutrition in FCs is more common compared to home-dwelling older people without a CR [4].

FCs’ impaired nutritional status could compromise the successful care of a CR for several reasons. Decreased nutritional status is related to decreased functional ability [2,5], such as decreased physical performance [5] and difficulties in daily activities such as shopping and cooking [6,7,8,9]. Impaired nutritional status is also related to decline in cognition [2,8] and depressive symptoms [10,11]. This association between nutritional status and depressive symptoms has also been shown in studies with older FCs [1,2,12]. In addition, oral health indicators are important factors of nutritional status [13]. It has also been shown that decreased nutritional status of FC is associated with decreased nutritional status of CR [1,2], and poor nutritional status of CR could increase the burden of care experienced by FCs [14].

Malnutrition and risk of malnutrition are associated with many undesirable factors in older people. Malnutrition is associated with longer hospitalization [15], costs of care [16], morbidity [17], and mortality [7,15]. Early detection and prevention of malnutrition could prevent frailty [18,19], the onset of disability [18], and reduce the length of hospital care [15]. Only few studies have studied the associations of functional ability [2], cognition [2], depression [2,20], and psychopathology [20] to nutritional status of FCs. Studies on the association of sense of coherence, quality of life, and oral health indicators with nutritional status of FCs are lacking.

It has been shown that there is a large variation in the dietary intake of older people, including non-optimal intakes [21]. However, there are no comprehensive data about the impact of deficiencies in nutrition on the nutritional status of FCs. Some data show that older people with lower nutritional status have poorer dietary intake than well-nourished older people [22]. By ensuring FCs’ adequate food intake, it would be possible to avoid impaired nutritional status.

Because good nutritional status is essential for a FC to be able to take care of a CR [2] we aimed to study the nutritional status and determinants of nutritional status of older FCs and the adequacy of the dietary intake of FCs compared to recommendations. The results of this study will help tailor nutrition support in this vulnerable population.

## 2. Materials and Methods

### 2.1. Participants

**Figure 1:**
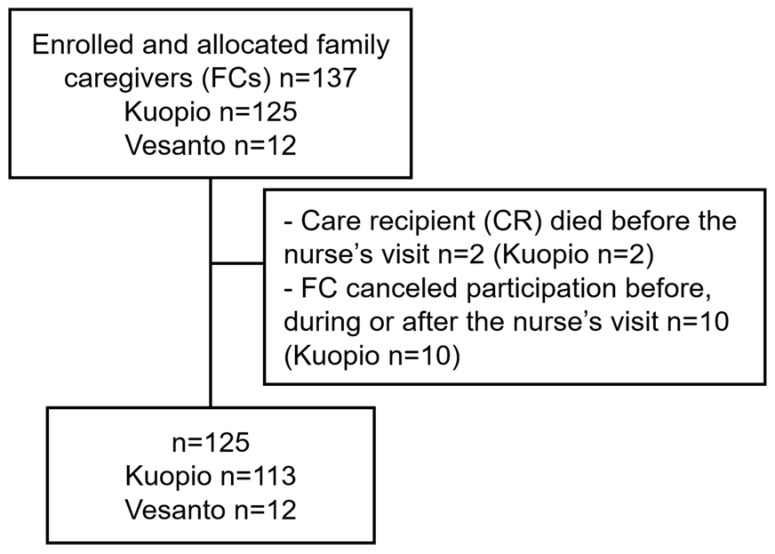
Flow chart of the study population.

The study participants consisted of 125 FCs living in the town of Kuopio and the municipality of Vesanto (Eastern Finland) (Figure 1). Participants were recruited from FC registers of Kuopio and Vesanto. All of the participants had a valid carer allowance (CA) during their participation in the study in the year 2019. In Finland, CA is granted by the municipality and includes benefits to the FC such as a taxable fee and a three-days of leave per month. It also includes necessary services for the CR, and informal care support services. FCs whose CR were receiving palliative care at baseline were excluded from the study. The service manager for older people in the town of Kuopio sent an invitation letter to all FCs whose CR was 65 years old or above. This is why it is possible to retrieve the age of a CR only. The invitation letter included a written invitation by the town of Kuopio to participate in the study, a written invitation by the researchers, and information about the study provided by the University of Eastern Finland and Kuopio University Hospital. In the municipality of Vesanto, all FCs whose CR was 65 years old or above were informed about the study by the service manager for older people of Vesanto. FCs who had given their consent received a written invitation letter and information of the study by the University of Eastern Finland and Kuopio University Hospital. The FCs included in the study were 60 years old or above, except two of them who were under the age of 60.

These data present the baseline data of both the intervention and the control groups of the LENTO (Lifestyle, Nutrition, and Oral Health in Caregivers) randomized controlled intervention study with individually tailored nutritional and oral health guidance for the FCs. The sample size was calculated based on plasma albumin concentration, with a 20% difference in plasma albumin concentration between the intervention and the control groups (power 0.80 and *p*-value 0.05). Based on this, a sample size 128 (*n* = 64 per group) was needed to demonstrate a statistically significant difference between the intervention and control groups.

### 2.2. Measurements

Both FCs and CRs were interviewed and measured during two home visits. A study nurse interviewed the FC regarding comorbidity using the Functional Comorbidity Index (FCI) [23], the use of daily prescription medication and daily use of supplementation, cognitive status according to the Mini Mental State Examination (MMSE) [24], depressive symptoms according to the Geriatric Depression Scale (GDS-15) [25], sense of coherence (SOC-13) [26], psychological distress according to General Health Questionnaire (GHQ-12) [27], quality of life according to the World Health Organization Quality of Life (WHOQOL)-BREF [28], Psychopathology of CR with Neuropsychiatric Inventory (NPI) [29], activities of daily living (ADL) according to Barthel Index [30], instrumental activity (IADL) with the Lawton and Brody Scale [31], and the CR’s comorbidity (FCI) and use of medication. The study nurse drew blood samples for measurements of concentrations of plasma albumin (P-Alb), plasma pre-albumin (P-Prealb) and blood hemoglobin (B-Hb) from both the FC and the CR. All laboratory analyses were analyzed by standard protocols at the Laboratory Centre of Eastern Finland ISLAB.

The study nurse gave a written introduction instructing the FC to keep a three-day food record. A clinical nutritionist and a dental hygienist made their visit one week after the study nurse’s visit. The clinical nutritionist assessed the FCs’ and CRs’ nutritional status with the Mini Nutritional Assessment (MNA) [32], which is a validated, simple, and inexpensive test [33]. The MNA consists of 18 brief questions and simple measurements: declined food use due to loss of appetite, digestive problems, chewing or swallowing difficulties; weight loss; mobility; psychological stress or acute disease; neuropsychological problems; body mass index (BMI); residency; medication use; pressure sores or skin ulcers; number of full meals; protein intake; fruit or vegetables consumption; fluid intake; autonomy of feeding; self-perception of nutritional status; self-perception of health; mid-arm circumference (MAC); calf circumference (CC). The clinical nutritionist measured the FC’s and CRs’ body weight, height, calculated BMI, measured MAC, and CC; physical performance was measured with a hand grip strength test (HGS) (Saehan Hydraulic Hand Dynamometer) [34], which was measured two times from both hands, but only the mean of the left hand was reported; a five times chair stand test was also carried out [35]. The nutritionist checked the FCs’ food record and vitamin D supplementation, as all other supplementations were included in the study nurse’s investigation. The clinical nutritionist checked those if necessary. For those who had not kept a food record, the clinical nutritionist performed a 24 h dietary recall. Dietary intake was calculated with the AivoDiet software (version 2.2.0.0, AivoDiet by Mashie, Turku, Finland).

The dental hygienist interviewed both FC and CR about the perception of dry mouth, swallowing, and chewing problems and performed a clinical oral examination for both. Self-reported problems in the mouth were assessed on a four-point scale (0 = no problems, 1 = 1 problem, 2 = 2 problems, 3 = 3 problems). The scale was formed from three dental hygienists questions “Can you eat dry bread or biscuit without drinking at the same time?”, alternatives were “yes” or “no”; “Can you chew hard or tough food, for example rye bread, meat or apple?”, alternatives were “without difficulties” or “yes, but chewing is difficult” or “not at all”; and “Do you have a perception of dry mouth?”, alternatives were “no” or “yes, sometimes” or “yes, continuously”.

### 2.3. Statistical Analyses

Mean with SD or number with percentage were calculated from the baseline characteristics. The normality of variables was checked with Shapiro–Wilk’s test. The normality of dependent variables of nutritional status was checked with Shapiro–Wilk’s test, histograms of the dependent variables, and histograms of standardized residuals of multivariate linear regression of the dependent variables. Univariate regression analysis was used to analyze associations between independent variables and the dependent variables of nutritional status. The independently associated variables were selected for multivariate linear regression analysis by a stepwise procedure to analyze significant determinants of nutritional status. Multicollinearity of multivariate linear regression analysis was checked with the tolerance and variance inflation factor (VIF). Differences between the nutritional status groups in dietary intake were analyzed by an independent samples *t*-test (normally distributed outcomes), Mann–Whitney’s U-Test (non-normally distributed outcomes), or Pearson’s Chi-square test. A *p*-value less than 0.05 was considered statistically significant. Data analyses were performed with the IBM SPSS Statistics software (v. 27, IBM Corp., Armonk, NY, USA).

## 3. Results

The demographics of the FCs (90 females and 35 males) are presented in Table 1. In our sample, the FCs’ mean age was 75 ± 7 years, and they were usually spouses or partners of the CR (89%); 10% were children of the CR, and two FCs had two CRs. The level of the CRs’ care needs can be seen in the fee classification. A total of 70% of the FCs had the first level of fee classification, which means that their CRs need care, support, or control many times in a day. The rest of the FCs had the second level of fee classification, and their CRs need special care or support round-the-clock. The FCs’ mean years of education were 11 ± 4, and their mean net income was 3139 ± 922 €/m. A total of 42% of the FCs had no self-reported problems in mouth; 36% had one problem, 14% had two problems, and 7% had three problems. The CRs had varying disease backgrounds; for example, 63% of the older CRs had dementia and 73% of them had Alzheimer’s disease (AD). Of the CRs, 37% had some other disease or impaired functional ability.

### 3.1. Nutritional Status

The clinical characteristics of the FCs are presented in Table 1. The nutritional status of the FCs was mainly at a good level, and the mean of the MNA scores was 25.5 ± 1.9. A total of 80% of the FCs were well-nourished (MNA scores ≥ 24.0), and 20% of the FCs were at risk of malnutrition (MNA scores 17–23.5). Similar results were found in plasma albumin and prealbumin concentrations. A total of 95% of the older FCs had good plasma albumin concentration (≥34 g/L), and 91% had good plasma prealbumin concentration (females ≥ 0.18 g/L, males ≥ 0.20 g/L).

#### 3.1.1. Determinants of MNA Scores

Significantly associated independent variables of the MNA scores are presented in Table 2. In the linear regression analyses, the FCI (*p* < 0.001), number of medications (*p* = 0.003), GDS-15 (*p* = 0.001), GHQ-12 (*p* = 0.009), WHO-QOL (*p* < 0.001), ADL (*p* = 0.039), B-Hb (*p* = 0.010), five times chair stand test (*p* = 0.010), self-reported problems in the mouth (*p* = 0.005), energy intake (*p* = 0.006), and protein intake (*p* = 0.013) were significantly associated with MNA scores of the FCs. There were no significant associations between any other variables and MNA scores. The multivariate linear regression analyses showed that significant determinants of lower MNA scores of the FCs were higher for FCI (*p* = 0.009), lower for WHOQOL-BREF (*p* = 0.001), and lower for energy intake (*p* = 0.020) (Table 2). After excluding two FCs who were under 60 years old from the univariate and multivariate analyses, there were no changes in the results.

#### 3.1.2. Determinants of Plasma Albumin Concentration

In the univariate linear regression analyses, GDS-15 (*p* = 0.027) and HGS (*p* = 0.030) were significantly associated with plasma albumin concentration of the FCs (Table 3). There were no significant associations between any other variables and plasma albumin concentration. In the multivariate linear regression analysis (*n* = 124) with selected independent variables, lower GDS-15 (*p* = 0.008) and lower HGS (*p* = 0.009) were significantly associated with lower plasma albumin concentration of the FCs (Table 3). After excluding two FCs under 60 years old from the univariate and multivariate analyses, age (B −0.063, 95% CI: −0.125, −0.001) was also significantly associated to plasma albumin concentration in the univariate analysis. However, it was not a significant factor in the multivariate analysis.

#### 3.1.3. Determinants of Plasma Prealbumin Concentration

In the univariate linear regression analyses, age (*p* = 0.011), BMI (*p* < 0.001), MAC (*p* < 0.001), CC (*p* = 0.002), HGS (*p* < 0.001), and self-reported problems in mouth (*p* = 0.013) were significantly associated with plasma prealbumin concentration (Table 4). The multivariate linear regression analysis (*n* = 125) with selected variables showed that significant determinants of lower plasma prealbumin concentration of the FCs were lower MAC (*p* < 0.001), lower HGS (*p* = 0.001), and higher number of self-reported problems in mouth (*p* = 0.029) (Table 4). After excluding two FCs under 60 years old FCs from the univariate and multivariate analyses, there were no changes in the results.

#### 3.1.4. Determinants of Blood Hemoglobin Concentration

In the linear regression analyses, age (*p* = 0.001), gender (*p* = 0.005), FCI (*p* = 0.012), number of medications (*p* = 0.048), MNA scores (*p* = 0.010), HGS (*p* < 0.001), 5 times chair stand test (*p* = 0.015), energy intake (*p* = 0.004), and protein intake (*p* = 0.009) were significantly associated with B-Hb (Table 5). The multivariate linear regression analysis (*n* = 122) with selected independent variables showed that higher age (*p* < 0.001), female gender (*p* < 0.001), lower MNA scores (*p* = 0.012), and lower energy intake (*p* = 0.013) were significant determinants of lower hemoglobin concentration of the FCs (Table 5). After excluding two FCs under 60 years old FCs from the univariate and multivariate analyses, the number of medications was not a significant factor for blood hemoglobin concentration in the univariate analysis. Upon removing the number of medications from the multivariate analysis, age (B −0.349, 95% CI: −0.614, −0.084), HGS (B 0.304, 95% CI: 0.090, 0.518), and energy intake (B 0.005, 95% CI: 0.001, 0.009) were significantly associated to blood hemoglobin concentration (adjusted R^2^ = 0.177, F = 9.470, *p* < 0.001).

### 3.2. Dietary Intake

Mean energy and protein intakes of the FCs are presented in Table 1. There was a significant difference between FCs with risk of malnutrition and well-nourished FCs in mean energy intake (1523 ± 437 kcal vs. 1760 ± 421 kcal, *p* = 0.014). The mean protein intake per actual body weight of the FCs was 0.96 ± 0.31 g/BW/day. There was no difference between FCs at risk of malnutrition and well-nourished FCs.

**Figure 2:**
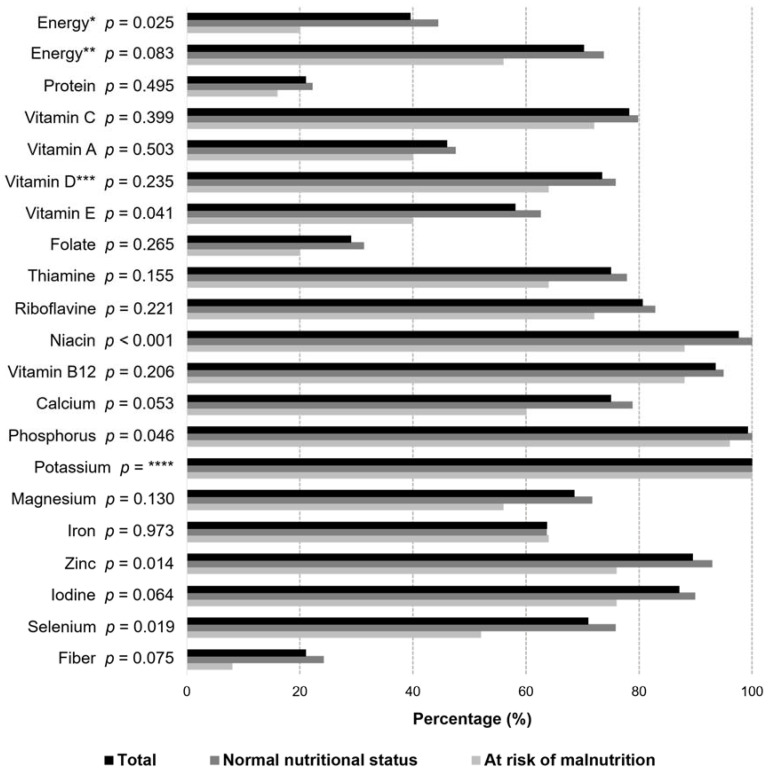
The proportion (%) of family caregivers (FC) in nutritional status groups whose energy and nutrient intake was recommended and statistical difference between groups by Pearson Chi-square test (*n* = 124). Energy recommendations: energy* ≥ 1700 kcal/day (females) or ≥ 2050 kcal/day (males), with PAL 1.4 (Nordic nutrition recommendations [36], energy** ≥ 1500 kcal/day [37]. Recommendations based on national nutrition recommendations (Nordic and Finnish nutrition recommendations [36,38]): protein ≥ 1.2 g/BW kg/day; vitamin A ≥ 700 RE/day (females) or ≥ 900 RE/day (males); vitamin C ≥ 75 mg/day; vitamin D ≥ 10 µg/day (age < 75) or ≥ 20 µg/day (age ≥ 75); vitamin E ≥ 8 mg/day (females) or ≥ 10 mg/day (males); folate ≥ 300 mg/day; thiamine ≥ 1.0 mg/day (females) or ≥ 1.2 mg/day (males); riboflavin ≥ 1.2 mg/day (females) or ≥ 1.3 mg/day (males); niacin ≥ 13 NE/day (females) or ≥ 15 NE/day (males); vitamin B12 ≥ 2.0 µg/day; calcium ≥ 800 mg/day; fiber ≥ 25 g/day; phosphorus ≥ 600 mg/day; potassium ≥ 3.1 g/day (females) or ≥ 3.5 g/day (males); magnesium ≥ 280 mg/day (females) or ≥ 350 mg/day (males); iron ≥ 9 mg/day; zinc ≥ 7 mg/day (females) or ≥ 9 mg/day (males); iodine ≥ 150 µg/day; selenium ≥ 50 µg/day (females) or ≥ 60 µg/day (males). *** Vitamin D intake from food and supplementation. **** No statistics are computed due to the constant variable.

As shown in Figure 2, there is a large proportion of FCs whose energy intake did not reach the recommended levels. Due to a lack of information about the daily physical activity level (PAL), energy intake was compared with two different recommendations. The first recommendation is based on the Nordic nutrition recommendations for older people [36], and the other is based on a study where 1500 kcal was considered the minimum of daily intake to provide the necessary intake of micronutrients [37]. When comparing the FCs’ energy intake to the Nordic recommendations, only 40% met the recommended intake. The majority (70%) of the FCs had adequate intake compared to the minimum intake of energy (≥1500 kcal/day). Figure 2 shows that majority of the older FCs had inadequate intake of protein (79%), vitamin A (54%), folate (71%), and fiber (79%) compared with the Nordic recommendations for older people [36]. About 25–40% of the FCs had inadequate intake of vitamin D, vitamin E, thiamine, magnesium, iron, and selenium. There were differences in the intake of energy and nutrients between well-nourished FCs and FCs at risk of malnutrition (Figure 2). Significant differences between the groups were found in the intake of energy, vitamin E, niacin, phosphorus, zinc, and selenium.

## 4. Discussion

Independent of the risk of malnutrition as assessed by the MNA the nutrient intake of FCs was commonly lower than recommended. Moreover, higher morbidity, poorer quality of life, and lower energy intake were associated with lower MNA scores in FCs. Furthermore, FCs’ lower HGS were related to lower plasma albumin concentration. Smaller MAC, lower HGS, and a higher number of self-reported problems in the mouth were associated with lower plasma albumin concentration. Finally, higher age, female gender, lower MNA scores, and lower energy intake were associated with a lower blood hemoglobin concentration in the older FCs. A high proportion of FCs had a low intake of protein, vitamin A, folate, and fiber.

### 4.1. Nutritional Status and Determinants

In our study, the proportion of well-nourished FCs (80.0%) was greater than that of earlier studies. Earlier studies have shown that there is variation in the nutritional status of older FCs. In a French study, 62.5% of older FCs of CRs with dementia were well-nourished [1]. Inferior results were found in study with older FCs of people with AD, where only 35.6% of FCs were well-nourished [2]. The clinical condition of participants could explain the difference between this present study compared to the previous. In this study, CRs were not limited to those with dementia or AD, and only 63% had diagnosed dementia. It could also be possible that FCs who are stressed or whose CRs’ situation is demanding did not participate in our intervention study because they see participating as too exhausting for them. This could have positively affected the results of nutritional status if the participants all consisted of FCs with better health conditions and life situations. The FCs in our study had a valid CA, which ensures taxable fees and regular health inspections for FCs. In Finland, FCs apply for the CA themselves; due to this, it is possible that exhausted FCs did not participate in the study. Additionally, FCs with poor health conditions cannot have CA. These facts can explain the differences in nutritional status compared to previous studies in other countries. Regarding the proportion of the older FCs whose plasma albumin and prealbumin concentration levels are good, over 90% had good nutritional status. That is close to the proportion of well-nourished participants according to the MNA scores and indicates that the participants in our study had better nutritional status compared to earlier studies [1,2].

Higher morbidity was significantly associated with decreased MNA scores of the FCs; this has been shown to be one predictor of malnutrition in earlier studies [17,39]. These older people with higher morbidity could have an association with a decline in cognition [40,41] and a decline in physical performance [39]. Additionally, there could be a decline in physical performance because of higher morbidity. It has also recognized that decline in physical performance is one consequence of impaired nutritional status [6,42]. Our study confirms that weak HGS predicts lower plasma albumin and prealbumin concentrations. Earlier studies have also shown that HGS could be a meaningful and easily executed assessment tool to support the measurement of the nutritional status of older people [43].

There was an association between better quality of life and better MNA scores. The association between quality of life and the nutritional status of older people has not been widely researched with the WHOQOL-BREF. One study with stroke patients found an association between the total scores of WHOQOL-BREF and nutritional status [44], and one study on an older population found an association between psychological well-being (one part of the WHOQOL-BREF) and nutritional status [45]. Throughout quality of life, nutritional status has a multilateral connection to comprehensive health, such as the burden of care [46], physical health [47,48], morbidity [49], and mortality [50], as well as its own connection to these health outcomes.

Mental health has been shown to be one remarkable predictor of nutritional status in our sample when lower GDS-15 scores were associated with better plasma albumin concentrations. Earlier depressive symptoms have shown to be a significantly negatively associated factor of MNA scores [1,2,10,11,12,17]. Our study’s contrary results could be explained by our sample’s low GDS-15 scores. There were also three outlier cases (high GDS-15 scores and high plasma albumin concentration), which could explain the contradictory result. However, higher GDS-15 scores and higher GHQ-12 scores were significantly associated with lower MNA scores of the older FCs in the univariate analyses. Consequently, it is not absolutely disqualified that poor mental health could decrease the MNA scores of older people.

Our study confirms earlier studies that found that there is a relationship between nutritional status and oral health problems [13,51]. More self-reported problems predict a lower prealbumin concentration, which could be explained by oral health problems affecting eating.

Considering the association between nutritional status and anthropometric measures, only MAC was significantly associated with plasma prealbumin concentration. Higher age and female gender were acceptably associated with lower hemoglobin concentration in our study [52]. Additionally, a lower hemoglobin concentration is associated with the risk of malnutrition, which was previously indicated in a large meta-analysis study [53].

Examining the relationship between magnitude of dietary intake and nutritional status, our study showed that a lower energy intake of the older FCs predicts lower MNA scores and lower hemoglobin concentration. Previously, there have been similar differences reported in dietary intake (as in our study) between nutritional status groups [22], but the association has not been studied in multivariate analyses.

### 4.2. Dietary Intake

A strength of our study is that a large number of FCs’ (84.8%) returned the three-day food record, which was checked by a clinical nutritionist. Moreover, 14.4% replied to a 24 h dietary recall performed by a clinical nutritionist, and there was only one participant (0.8%) who did not participate.

The older FCs’ had the same significant difference in mean intake of energy between nutritional status classification groups. Many studies have shown that energy and nutrient intakes differ widely in older people [21,54,55]. For this reason, we considered the sufficiency of dietary intake. In our study, a large number of the older FCs had an inadequate intake of energy according to the Nordic nutrition recommendations [36], especially FCs with a risk of malnutrition. An inadequate intake of energy according to recommendations was also found in FCs with AD spouses [3] and home-living older people [55]. A minimum daily intake of food totaling 1500 kcal enables an adequate intake of nutrients [37]. The mean energy intake of older people usually achieved this minimum in earlier studies [3,54,55], and in our study only 70% of the older FCs achieved the recommended intake. An alarming proportion of the older FCs had a low intake of protein (79%), fiber (79%), folate (71%), and vitamin A (54%). Therefore, the consumption of protein-rich products, wholegrain products, and vegetables was insufficient in a large number of the older FCs. Additionally, 25–40% of the older FCs had deficiencies in consumption of vegetable oils, nuts, seeds, liver, and use of vitamin D supplementation because of inadequate intakes of vitamin D, vitamin E, thiamine, magnesium, iron, copper, and selenium. Low energy and nutrient intake could be due to, e.g., poor appetite [56] or the FCs’ relationship to food and overweight [57].

In our study, low protein intake corresponds to the findings of earlier studies in older people. Generally, the mean protein intake has varied from 0.86 to 1.0 g/BW kg/day in earlier studies in older people [3,22,54,55]. In only one study, a home-dwelling group of older people achieved 1.2 g/WB kg/day intake of protein as recommended by the Nordic nutrition recommendations [55]. Inadequate intake of protein could lead to frailty [18,19,58], loss of lean mass [18], and it could also decelerate recovering from illnesses [18].

There was a conflict in the results between sufficient protein intake and plasma albumin concentration. Over 90% of the FCs had good plasma albumin concentration, but only 20% of the FCs had sufficient protein intake. Although plasma albumin concentration is widely used as a marker of protein intake and malnutrition, the association is not unequivocal, and it is not reliable by itself [59]; e.g., dehydration can increase plasma albumin concentration [60].

### 4.3. Strengths and Limitations

The strengths of the present study include the multi professional study group, its population-based design, and the validated methods in older people. The data on MNA and food records were collected/checked by a clinical nutritionist, which improves the study’s reliability. Our study was carried out during home visits, which improved the possibility for participation. However, some of the FCs may have seen participating in the intervention study as burdensome. This could have negatively affected to our sample size and distorted the results if the participants all consisted of FCs in good situations.

## 5. Conclusions

Higher morbidity, poorer quality of life, lower energy intake, weaker physical performance, smaller MAC, and a higher number of self-reported problems in the mouth were associated with decreased nutritional status in older FCs. It was common for FCs to have lower than recommended intakes of energy and several nutrients, independent of the risk of malnutrition as assessed by the MNA. It is important to follow both the nutritional statuses and dietary intakes of older FCs regularly to identify those with lower than recommended nutrient intakes and to further avoid poor nutritional statuses from hampering their ability to serve as FCs.

## Figures and Tables

**Table 1:** Demographics and clinical characteristics of the older family caregivers (FC), mean and standard deviation (SD), or *n* and percentage (%) (*n* = 125).

	Variables	Mean ± SDor *n* (%)
Demographics	Age (year)	74.6 ± 7.3
	Female, *n* (%)	90 (72.0)
Clinical Characteristics	FCI	1.9 ± 1.5
	Number of medications	5.3 ± 3.6
	P-Alb (g/L) ^1^	37.6 ± 2.4
	P-Alb 34 (g/L) or more, *n* (%) ^1^	118 (95.2)
	P-Prealb (g/L)	0.25 ± 0.04
	P-Prealb females 0.18 (g/L) or more, males 0.20 or more, *n* (%)	114 (91.2)
	B-Hb (g/L) ^3^	135 ± 10
	MNA scores	25.5 ± 1.9
	17–23.5 points, *n* (%)	25 (20.0)
	˃23.5 points, *n* (%)	100 (80.0)
	BMI (kg/m^2^)	28.4 ± 5.6
	23–29 kg/cm^2^, *n* (%)	63 (50.4)
	>29 kg/cm^2^, *n* (%)	45 (36.0)
	MAC (cm)	32.5 ± 4.5
	CC (cm)	38.5 ± 3.9
Functional Characteristics	MMSE	26.4 ± 3.0
	GDS-15	3.0 ± 2.4
	GHQ-12	12.5 ± 5.2
	WHOQOL-BREF ^1^	93.4 ± 9.4
	ADL	98.0 ± 3.5
	IADL	6.8 ± 0.5
	HGS (kg)	24.8 ± 8.4
	5 times chair stand test (s) ^1^	13.1 ± 4.3
Other Characteristics	SOC-13	61.7 ± 6.7
	NPI ^2^	3.5 ± 2.7
Dietary intake characteristics	Energy intake (kcal/day) ^1^	1712 ± 433
	Protein intake (g/day) ^1^	69.6 ± 19.3

SD = standard deviation, FCI = Functional Comorbidity Index, P-Alb = plasma albumin, P-Prealb = plasma prealbumin, B-Hb = blood hemoglobin, MNA = Mini Nutritional Assessment, BMI = body mass index, MAC = mid-arm circumference, CC = calf circumference, MMSE = Mini Mental State Examination (range 0–30), GDS-15 = Geriatric Depression Scale (range 0–15), GHQ-12 = General Health Questionnaire (range 0–36), WHOQOL-BREF = the World Health Organization Quality of Life-BREF (range 0–130), ADL = Barthel Index (range 0–100), IADL = Instrumental Activities of Daily Living (range 0–8), HGS = hand grip strength, SOC-13 = sense of coherence (range 13–91), NPI = neuropsychiatric inventory (range 0–144). ^1^ *n* = 124; ^2^ *n* = 117; ^3^ *n* = 123.

**Table 2:** Significantly (*p* < 0.05) associated independent variables of Mini Nutritional Assessment (MNA) scores of the older family caregivers (FC) according to univariate linear regression analysis (*n* = 125) and multivariate linear regression by stepwise procedure (*n* = 120).

	Univariate	Multivariate ^3^
Variables	B (95% CI)	B (95% CI)
FCI	−0.430 (−0.648, −0.211)	−0.283 (−0.492, −0.073)
Number of medications	−0.143 (−0.235, −0.051)	
GDS-15	−0.243 (−0.380, −0.107)	
GHQ-12	−0.087 (−0.152, −0.022)	
WHOQOL-BREF ^1^	0.055 (0.027, 0.083)	0.045 (0.018, 0.072)
ADL	0.102 (0.005, 0.199)	
B-Hb (g/L) ^2^	0.043 (0.010, 0.075)	
5 times chair stand test (s) ^1^	−0.104 (−0.182, −0.026)	
Self-reported problems in mouth	−0.529 (−0.893, −0.164)	
Energy intake (kcal/day) ^1^	0.001 (0.000, 0.002)	0.001 (0.000, 0.002)
Protein intake (g/day) ^1^	0.022 (0.005, 0.040)	

B = beta, CI = confidence interval, FCI = Functional Comorbidity Index, GDS-15 = Geriatric Depression Scale, GHQ-12 = General Health Questionnaire, WHOQOL-BREF = the World Health Organization Quality of Life-BREF, ADL = Barthel Index, B-Hb = blood hemoglobin. ^1^ *n* = 124; ^2^ *n* = 123; ^3^ *n* = 120. Adjusted for FCI, number of medications, GDS-15, GHQ-12, WHOQOL-BREF, ADL, B-Hb, 5 times chair stand test, self-reported problems in mouth, energy intake, protein intake; R^2^ = 0.197; *t*-test = 10.739; *p* < 0.001.

**Table 3:** Significantly (*p* < 0.05) associated independent variables of plasma albumin concentration (P-Alb) of the older family caregivers (FC) by univariate linear regression analysis (*n* = 124) and multivariate linear regression by stepwise procedure (*n* = 124).

	Univariate	Multivariate ^1^
Variables	B (95% CI)	B (95% CI)
GDS-15	0.195 (0.022, 0.368)	0.234 (0.063, 0.405)
HGS (kg)	0.055 (0.005, 0.105)	0.067 (0.017, 0.116)

B = beta, CI = confidence interval, P-Alb = plasma albumin, GDS-15 = Geriatric Depression Scale, HGS = hand grip strength. ^1^ Adjusted for GDS-15, HGS; R^2^ = 0.078; F = 6.201; *p* = 0.003.

**Table 4:** Significantly (*p* < 0.05) associated independent variables of plasma prealbumin concentration (P-Alb) of the older family caregivers (FC) according to univariate linear regression analysis (*n* = 125) and multivariate linear regression by stepwise procedure (*n* = 125).

	Univariate	Multivariate ^1^
Variables	B (95% CI)	B (95% CI)
Age (year)	−0.001 (−0.002, 0.000)	
BMI (kg/m^2^)	0.003 (0.001, 0.004)	
MAC (cm)	0.004 (0.002, 0.005)	0.003 (0.002, 0.005)
CC (cm)	0.003 (0.001, 0.005)	
HGS (kg)	0.002 (0.001, 0.003)	0.001 (0.001, 0.002)
Self-reported problems in mouth	−0.011 (−0.019, −0.002)	−0.008 (−0.016, −0.001)

B = beta, CI = confidence interval, P-Alb = plasma albumin, BMI = body mass index, MAC = mid-arm circumference, CC = calf circumference, HGS = hand grip strength. ^1^ Adjusted for age, BMI, MAC, CC, HGS, self-reported problems in mouth; R^2^ = 0.226; F = 13.061; *p* < 0.001.

**Table 5:** Significantly (*p* < 0.05) associated independent variables of blood hemoglobin concentration (B-Hb) of the older family caregivers (FC) according to univariate linear regression analysis (*n* = 123) and multivariate linear regression by stepwise procedure (*n* = 122).

	Univariate	Multivariate ^1^
Variables	B (95% CI)	B (95% CI)
Age (year)	−0.406 (−0.651, −0.162)	−0.492 (−0.719, −0.264)
Gender	5.707 (1.736, 9.678)	6.871 (3.192, 10.550)
FCI	−1.554 (−2.757, −0.352)	
Number of medications	−0.512 (−1.019, −0.005)	
MNA scores	1.246 (0.298, 2.193)	1.144 (0.255, 2.033)
HGS (kg)	0.420 (0.212, 0.627)	
5 times chair stand test (s)	−0.533 (−0.962, −0.103) ^2^	
Energy intake (kcal/day)	0.006 (0.002, 0.011) ^2^	0.005 (0.001, 0.009)
Protein intake (g/day)	0.128 (0.033, 0.224) ^2^	

B = beta, CI = confidence interval, P-Alb = plasma albumin, FCI = Functional Comorbidity Index, MNA = Mini Nutritional Assessment, HGS = hand grip strength. ^1^ *n* = 122. Adjusted for age, gender, FCI, number of medications, MNA scores, HGS, 5 times chair stand test, energy intake, protein intake; R^2^ = 0.252; F = 11.098; *p* < 0.001. ^2^ *n* = 122.

## Data Availability

Based on the EU regulations we cannot make data freely available. Upon request the authors will provide the data, e.g., for meta-analyses.
